# Oncologic drug repository programs in the United States: a review and comparison

**DOI:** 10.1093/haschl/qxae031

**Published:** 2024-03-06

**Authors:** Natalie K Heater, Sheetal Kircher, Christine Weldon, Julia Trosman, Al Benson

**Affiliations:** Department of Medicine, Northwestern Memorial Medical Center, Chicago, IL 60611, United States; Department of Hematology and Oncology, Robert H. Lurie Comprehensive Cancer Center of Northwestern University, Chicago, IL 60611, United States; Center for Business Models in Healthcare, Northwestern University Feinberg School of Medicine, Chicago, IL 60611, United States; Center for Business Models in Healthcare, Northwestern University Feinberg School of Medicine, Chicago, IL 60611, United States; Department of Hematology and Oncology, Robert H. Lurie Comprehensive Cancer Center of Northwestern University, Chicago, IL 60611, United States

**Keywords:** medication waste, medication affordability, high-cost medication, drug repository program, oral anticancer drug cost

## Abstract

As cancer affects 40% of all Americans during their lifetime, the financial burden of cancer care represents a significant contribution towards the overall cost of health care in the United States. Cancer drug repository programs offer a unique solution for patients who have limited financial ability to access medications while reducing medical waste. We reviewed all state legislation in the United States regarding cancer drug repository programs. Five states have oral anticancer drug (OACD)–specific drug repository programs, while 28 states have generalized drug repository programs. Iowa's statewide, mail-order OACD repository program is the preeminent example of an effective and efficient program, which should be replicated across the country. Many states have passed legislation allowing for drug repository programs but have struggled to translate such legislation into active programs due to lack of funding and management. We offer recommendations across policy, manufacturing, institutional, health care professional, and patient domains in order provide optimal patient care.

## Introduction

The cost of health care in the United States is unsustainable. While new technologies extend the lifespan of patients with cancer, the cost of cancer care is rising precipitously. Cancer care is estimated to cost $246 billion annually by 2030.^1^ Reducing health care costs is a fundamental issue in health care policy.

The rise of oral anticancer drugs (OACDs) is changing the landscape of oncologic treatment. In 2020, OACDs constituted 67% of all newly approved anticancer therapies^2^ and are often used in the first line of treatment.^3,4^ Oral anticancer drugs offer advantages over parenteral treatments, including patient convenience and reduction in the number of outpatient visits^5^; however, OACDs may come with increased out-of-pocket (OOP) cost for patients.^6^

Studies have found that as many as 40% of patients with cancer spend their life savings in the first 2 years of treatment, with a least a portion of this burden due to the cost of cancer therapies and supportive medications.^7^ This often-unattainable cost of cancer treatments is attributed to factors such as high research and development costs, high prices set by manufacturers, the effect of drug policy and patent laws, intermediary costs such as those imposed by pharmacy benefit managers, and difficulties accessing payment assistance.^8^ In 2022, patients taking OACDs were responsible for a median annual OOP cost of $12 979 per year.^9^

Drug repository programs allow for the recycling and re-distribution of unused and unwanted medications to qualified patients. The goal of drug repository programs is to reduce medication waste and improve access of high-cost medications to patients with limited financial ability to pay for these medications. The first state-sponsored drug repository program was created in Georgia in 1997.^10^ To date, there is no federal drug repository program. The Food and Drug Administration (FDA), the American Medical Association, and National Association of Boards of Pharmacy support medication redistribution systems.^10^

Given the pervasive use of high-cost OACDs, variability in insurance coverage, and high volume of medication waste, OACD repository programs provide enticing opportunities to improve patient access to OACDs. Many OACDs are classified as Risk Evaluation and Mitigation Strategies (REMS) medications by the FDA, which is a program designed to provide an additional level of safety for medications with serious side effects. Many generalized drug repository programs exclude REMS medications due to increased operational costs to ensure these safety measures are met, highlighting the need for an OACD-specific program to operate under the guidance of specialty trained staff. To the authors’ knowledge, no comprehensive analysis of OACD repository programs in the United States has been published in the literature to date. This article aims to highlight and compare existing OACD drug donation programs in order to develop potential best practices for the expansion of programs.

### Insurance coverage and patient-assistance programs for OACDs

More than half of new cancers occur among Americans aged 65 years and older, most of whom obtain their primary insurance coverage through Medicare.^11^ Medicare, which is administered by the Centers for Medicare and Medicaid Services, finances multiple components of cancer care. Private insurers follow a similar coverage structure and typically separate hospital, outpatient, and pharmacy benefits. Prescription coverage through Medicare Part D has 4 phases (deductible, initial coverage, coverage gap, and catastrophic coverage) with varying OOP cost for each phase. For 2023, after the patient meets their deductible, they will be responsible for the co-pay specific to their prescribed medications until the total retail cost of their medications exceeds $4660 (initial coverage). Subsequently, the patient then reaches the coverage gap, during which they are responsible for 25% of the retail cost of their medications until the true OOP cost (defined as deductible + 25% copay from initial coverage + 95% of retail cost in coverage gap) reaches $7400. At this point, the patient enters the catastrophic coverage phase and is responsible for only 5% of the cost of their medications.^12^ For many patients with cancer, the initial prescription often exceeds the deductible and initial coverage phases, landing patients in the coverage gap or catastrophic coverage where there is no limit to OOP costs.

Patients with commercial insurance may benefit from grant funding, manufacturer assistance programs, or patient-assistance programs (PAPs) that can help to decrease OOP cost. These resources are not available to Medicare beneficiaries. Utilization rates of PAPs for OACD prescriptions in the literature vary, with reported rates from 36% to 54%.^13,14^ Patient-assistance programs are associated with decreased OOP cost, from a median of $330 to $25, with the tradeoff of increased time to medication acquisition and higher medical office administrative needs.^14^

### Medication waste

At the same time as patients struggle to afford medication costs, unused and unexpired medications worth $11 billion (list price) go to waste each year,^15^ with an unknown proportion due to OACDs. Patients discontinue OACDs most often secondary to disease progression (28%), death (22%), or adverse events of therapy (7%),^16,17^ with waste occurring in 34%–41% of patients. Waste due to dose reduction or discontinuation of OACDs costs a median of $1750 per patient.^18^

## Data and methods

We reviewed all state legislation in the United States regarding cancer drug repository programs. We then searched each individual state government website for proposed and enacted statutes. See Appendix 1 for the full search protocol. States with OACD-specific repository programs were included for further analysis. States were queried via phone and email to reach program coordinators. If no such contact information existed, an analysis of their publicly available website was conducted. Our internal database was compared with existing databases including the National Conference of State Legislatures,^19^ the 2020 Position Statement on Drug Repository Programs published by the American Society for Clinical Oncology (ASCO),^10^ and the Supporting Initiative to Redistribute Unused Medicine (SIRUM)^20^ to ensure accuracy of results. Variables collected included program titles, date of inception, state act citation, regulating entity, number of participants, donor eligibility, recipient eligibility, cost to recipient, and ability to track outcomes.

## Results

### OACD-specific programs

As of September 2023, 5 states (Florida, Iowa, Michigan, Montana, and Nebraska) have active OACD-specific repository programs in addition to generalized drug repository programs, while Nevada and Pennsylvania's OACD-specific programs are no longer active. A summary of OACD-specific programs is outlined in Table 1.

**Table 1.** OACD-specific repository programs.

State	Year of inception	State legislature code	Title	Regulating entity	Number of participants	Donor eligibility	Open or closed system	Recipient eligibility	Outcome tracking	Cost to recipient
Florida	2020	61N-1.026	Cancer Drug Donation Program	Department of Business and Professional Regulation	Unknown	Health care facilities, hospitals, and pharmacies	Closed	Uninsured or do not qualify for third-party insurance coverage, Medicaid or Medicare, or any other state or federal assistance programs	No	$15.00 or 300% of the Medicaid dispensing fee, whichever is less
Iowa	2007	641 IAC 109.1	Cancer Donation Medication Program	Iowa Department of Public Health with SafeNetRx, nonprofit	One centralized repository with mail-order system to distribute to entire state	Any person or organization can donate medication or supplies	Open	Uninsured or below 200% of Federal Poverty Level	Yes	None
Michigan	2006	333.17775	Cancer Drug Repository Program and Utilization of Unused Prescription Drugs Program	Department of Licensing and Regulatory Affairs	9 participating pharmacies	Individual donors, manufacturers, pharmacies, and clinics	Open	Uninsured or eligible for Medicare or Medicaid, or otherwise lacks reasonable means to purchase drugs	No	$5.00 or 250% of the Medicaid dispensing fee, whichever is less
Montana	2009	37-71401 to 37-71408	Cancer Drug Repository	Board of Pharmacy	Does not track participating long-term-care facilities	Long-term-care facilities	Closed	“Qualified patients”	No	Unknown
Nebraska	2003	71-2422 to 71-2430	Cancer Drug Repository	Department of Health and Human Services	75 participating locations (hospitals, physician offices, pharmacies)	Any person or entity, including, but not limited to, a cancer drug manufacturer or health care facility; does accept injection drugs if no temperature storage requirements	Open	Anyone with cancer, has a prescription for the medication and is unable to afford it	No	Not to exceed Medicaid dispensing fee
Nevada	2010	453B.010 to 453B.240	Cancer Drug Donation Program	State Board of Pharmacy	0 participating locations	Pharmacies, medical facilities, health clinics, or health care provider	Closed	Eligible patients	No	May charge a handling fee
Pennsylvania	2013	49 27927.501 to 27.506	Cancer Drug Repository Program	Board of Pharmacy	0 participating pharmacies	Pharmacy, facilities, drug manufacturers or wholesale drug distributor pharmacy, health care facility, drug manufacturer, or wholesale drug distributor	Closed	Uninsured or underinsured so that the coverage limits prevent the patient from obtaining cancer drugs and does not meet eligibility requirements for State Medical Assistance Program; income limit not to exceed 350% of Federal Poverty Level for family size	No	May charge a handling fee

Abbreviation: OACD, oral anticancer drug.

In general, the programs require medications to be unexpired and in manufacturer-sealed packaging, must be inspected by a pharmacist, and must be received by patients with financial need. State regulations vary regarding how close to their expiration date OACDs can be donated, where donated medications are stored, availability of state funding, and metrics to track success. Most programs are regulated by state agencies, such as the Board of Pharmacy or Department of Health.

Iowa, Michigan, and Nebraska are “open systems,” meaning that medications that have been distributed to patients can be donated back into the drug supply as long as they pass inspection by a pharmacist. In contrast, a “closed system” does not allow medications that have been already distributed to patients to re-enter the system; drugs must have been maintained under a health care practitioner to be eligible for redistribution.^10^ Florida, Montana, Nevada, and Pennsylvania are closed systems.

Iowa's OACD-specific repository program will be highlighted in detail as its statewide distribution system, ability to track outcomes, and guaranteed budget make it an ideal program to replicate in other states. In 2007, the Iowa Legislature provided for a statewide, mail-order drug repository system that was the first of its kind in the United States. This program is administered by SafeNetRx, a nonprofit wholesale pharmacy, in conjunction with the Department of Public Health. Although SafeNetRx has accepted OACDs since inception, a 2016 cancer-specific partnership with cancer centers began with the hopes to increase OACD donation. From 2016 to 2022, over 84 000 doses of chemotherapy worth $15 000 000 have been distributed to patients in need.^21^ Notably, SafeNetRx has statewide budgetary support and a centralized processing center for all donations. With the help of 6 full-time pharmacists and volunteers who donate 2000 hours per year, SafeNetRx can mail medications to patients across the entire state within 3–5 business days. The ability of SafeNetRx to track donations and publicly report on their success drives further success.

Nebraska and Michigan also have operational, open-system OACD-specific drug repository programs, but rely on participating pharmacies to decide which OACDs to accept and redistribute. Nebraska has 75 participating pharmacies, while Michigan has 9.^22,23^ Unlike Iowa, the programs in Nebraska and Michigan do not report outcomes, limiting the ability to quantify their reach and impact.

The OACD-specific closed systems have had limited success. Montana's cancer drug repository allows for redistribution of cancer medications within nursing homes but does not report on participating locations or outcomes. Nevada’s and Pennsylvania's programs are defunct, while Florida's program could not be assessed as operational or non-operational.

### Generalized drug repository programs

While only 5 states have statewide active OACD-specific drug repository programs, 44 states and Washington, D.C., have passed legislation allowing for a generalized drug repository program. However, only 28 states have materialized legislation into operational programs (AZ, CA, CO, FL, GA, IL, IA, KS, KY, LA, MD, MI, MN, MT, NE, NM, NC, ND, OH, OK, OR, SD, TN, TX, VA, WA, WI, and WY). A state is considered to have an operational program if there is at least 1 participating site (pharmacy, medical facility, clinic, etc) in the state. Notably, an operational program does not mean that individuals across the state will have access to that site, that a particular drug is accepted at that site, or that an accepted drug will be available at that site. This applies to both medications broadly and OACDs specifically. A common exception to drugs accepted by repository programs includes drugs classified as an REMS drug by the FDA. Each individual site may impose additional restrictions. Overall, there is no standardized procedure for acceptance or redistribution of OACDs.

Sixteen states (AL, AR, CT, ID, IN, MS, MO, NV, NH, NJ, NY, PA, RI, UT, VT, and WV) have passed legislation authorizing drug repository programs but do not have active programs. Six states (AK, DE, HI, ME, MA, and SC) have not passed legislation establishing a drug repository program. Residents of these states may be eligible to donate medications via mail to a nationalized nonprofit such as SIRUM but do not access to any state-sponsored resources.

**Figure 1.**
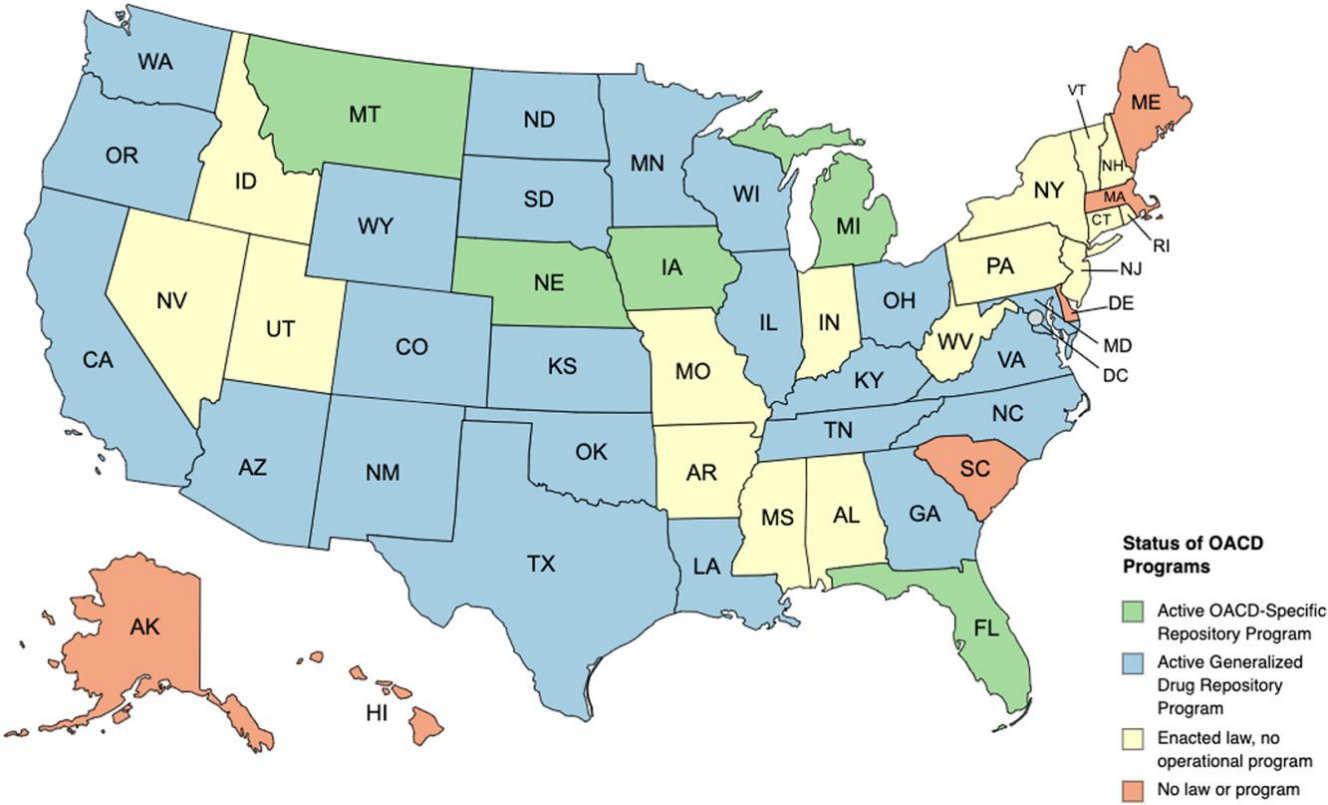
Summary of drug repository programs in the United States. Abbreviation: OACD, oral anticancer drug. Created with mapchart.net.

Figure 1 summarizes our findings regarding the status of drug repository legislation and programs in all 50 states.^24^ States with active/operational OACD-specific drug repository programs are colored green, states with active generalized drug repository programs are colored blue, and states with legislation but no active generalized drug repository programs are colored yellow. The 6 states without legislation or an active program are colored red.

SIRUM is a nonprofit dedicated to expanding access to medications through drug repository programs, including OACDs. SIRUM is the largest redistributor of surplus medications in the United States, processing 650 000 prescriptions worth $58 000 000 in 2022.^20^ SIRUM helps individuals donate from most states across the country and facilitates donations to their community partners in 40 states. Their broad network allows SIRUM to overcome local supply issues by redistributing medications across their community partners. Minnesota, Ohio, Georgia, Colorado, and California have all partnered with SIRUM to provide infrastructure support for their own state-specific programs.

Notably, Minnesota modeled its Medication Repository Program after Iowa's SafeNetRx, with the support of SIRUM. The Minnesota Board of Pharmacy partnered with RoundtableRx, a nonprofit wholesale pharmacy, to implement its drug recycling program, including OACDs, in 2020. Residents, pharmacies, and long-term-care facilities can donate to the program. RoundtableRx is run with support from SIRUM and secured state funding that will begin in 2024.^25^ RoundtableRx delivers medications to 23 clinics or pharmacies across the state, which then dispense the medications to patients. This ensures consistency across all participating sites. Upcoming initiatives include implementing a mail-order system to improve access. As of September 2023, over 44 000 medications have been recycled worth over $1 600 000.^25^ Although they accept some OACDs, they do not track OACDs separately from medications generally.

The Ohio Drug Repository Program has 40 actives sites across the state in an open system, operating in a partnership between the Ohio Board of Pharmacy and SIRUM.^26^ An unknown number of those 40 sites allow OACDs to be recycled. A 2023 expansion of the drug repository program now allows for medications that are no longer in their original, sealed packaging to be donated, provided the medications pass visual inspection by a licensed pharmacist.^27^

In 2020, a pilot program at The Ohio State University Comprehensive Cancer Center (OSUCCC) in conjunction with the Ohio Board of Pharmacy aimed to define metrics of a successful OACD repository program. Patients were deemed eligible by financial counselors within their PAP and offered the opportunity to participate if the needed medication was available. A media campaign targeted at oncology patients raised awareness of the program. After a review of OACDs consistently in demand for payment assistance and with an expected suitable donation-based supply, OSUCCC piloted a repository program for capecitabine and temozolomide,^28^ which is still ongoing and expanding to offer other OACDs.

## Discussion

Efforts to decrease health care costs are an important policy issue. Cancer drug repository programs offer a unique solution for patients who have limited financial ability to access lifesaving medications while reducing medical waste. Iowa's statewide, mail-order OACD repository program stands out as a uniquely effective and efficient program that should be replicated across the country. Many states have passed legislation allowing for drug repository programs, but have struggled to translate legislation into active, successful programs due to lack of funding and management. SIRUM offers an alternative to state-specific programs by creating a nationalized approach.

We conclude that components of success include a supportive infrastructure with renewable sources of funding, access to all state residents via mail orders, ability to track outcomes, and marketing the program to potential medication donors. In contrast, states without these resources are limited in their impact as many residents may not have geographic proximity to a participating pharmacy (eg, Michigan) or may not be eligible for the program (eg, closed system within long-term-care facilities in Montana). As exemplified by Nevada and Pennsylvania, without renewable resources the program may become defunct altogether.

The ASCO's most recent position statement supports the expansion and implementation of OACD repository programs, as long as they do not incur a cost to the patient beyond standard pharmacy dispensing fees.^10^ The statement was updated in 2022 to advocate for open systems after the National Board of Pharmacy and the American Medical Association endorsed the use of open, rather than closed, systems for drug redistribution.^10^ At this time, the FDA does not yet endorse the use of an open redistribution system, which we urge the FDA to reconsider.

Based on our findings, we know that change is needed in multiple different domains in order to expand and improve current practices involving OACD repository programs. We developed the following recommendations, which are outlined in Table 2.

**Table 2.** Summary of best practices for OACD program expansion.

Stakeholder	Recommendations
Policymakers	Revise existing drug repository legislation to include OACDs Existing and new legislation should contain at a minimum the following provisions: Obtain designated renewable state funding Designate state agency responsible for program Mandate tracking of common outcomes data Allow for an open distribution system Prioritize recipient eligibility based on income, insurance status, and access to other payment-assistance programs Mandate low or no-cost to recipients Encourage the FDA to endorse the use of an open redistribution system
Manufacturers	Package medications in single unit dose packaging
Institutions	Standardize donation policies and procedures across the state Recruit participant locations from across the state Establish a network of communication between sites to redistribute OACDs between sites, or create a centralized repository with mail-order system to ensure access Educate medical staff of program and procedures for donation
Health care professionals	Educate clinicians regarding program and procedures for donation Involve pharmacist and pharmacy technicians to inspect each donated medication and ensure compliance with donation standards
Patients	Educate patients and their families of program and procedures for donation

Abbreviations: FDA, Food and Drug Administration; OACD, oral anticancer drug.

Policymakers should implement new legislation to expand existing and create new OACD repository programs. The OACD repository programs should be separated from generalized drug repository programs given their high cost and toxic effects. The OACD-specific programs may allow for fewer restrictions to redistribution based on REMS classification, which are often necessary in generalized OACD programs to keep operational costs low. Legislation must include a renewable source of funding and stipulations for tracking metrics such as participating sites, number of donations, and number of prescriptions filled. It should also ensure that medications disbursed to patients are able to be redistributed in an open system, accepting donations from patients, families of patients, pharmacies, drug manufacturers, and health care facilities. Finally, in ensuring equity, medications should be prioritized for patients with limited financial means and should be provided at low or no cost.

Manufacturers should produce medications in individual sealed packaging, such as blister packs. More information is needed before endorsing acceptance of donated medication no longer in original, sealed packaging as this may put undue stress on pharmacy staff to inspect and approve these donations.

Institutions should ensure that their programs are standardized and scalable across the state. A mail-order system offers the advantage of ensuring delivery to all residents, regardless of proximity to participating pharmacies. Finally, education to potential partners including clinical teams and patients is imperative to connect donors and recipients.

Many high-cost, non-OACD medications are included within generalized drug repository programs; OACD medications are often excluded from generalized repositories due to their REMS status. To the authors’ knowledge, no other drug repository programs exist for other non-OACD REMS medications, which include anti-psychotics, anti-seizure medications, opiate medications, and biologics used in many medical specialties. A repository program to include all REMS medications could be incorporated into a “specialty pharmacy” drug repository program to address this need. However, OACDs represent the majority of high-cost REMS medications and should be prioritized in repository programs in order to lower health care costs.

An important caveat to this research is the possibility of buyback programs from insurance companies themselves, in which patients could return unused medications back to the pharmacy and receive a refund in return. This strategy was piloted by the Department of Veteran Affairs, which offered veterans $5 for each unused opioid pill that was prescribed after ambulatory surgery.^29^ If this model was scaled across all insurance companies, it would render drug repository programs superfluous. However, in the authors’ view, this is an unlikely solution in the private insurance market given the potential financial losses that private insurance companies would suffer. Further research on buyback programs is needed to demonstrate benefit across financial and public health domains.

Additionally, given that redistributed medications may be subject to a processing fee for recipients in the current model, one could argue that these medications are paid for twice. Further implications of this, such as the medical loss ratio for insurance companies, should be examined.

Our research was limited by the availability of publicly available information on drug repository programs. No up-to-date resource of existing programs is available, with often-conflicting information found in available resources. There is no standardized process for reporting how many participating sites exist in each state, or whether that participating site accepts certain medications including OACDs. Every effort was made to accurately represent up-to-date and accurate information. The authors acknowledge that this lack of reporting hinders the expansion of drug repository programs across the United States.

## Conclusion

Oral anticancer drugs are widely utilized but contribute to rising health care costs. Barriers in insurance coverage and medication waste make OACD repository programs an important aspect of high-value, accessible, and affordable cancer care. Five states have active OACD repository programs with varying levels of success. Our analysis suggests that changes across policy, manufacturing, institutional, health care professional, and patient domains are needed to optimize OACD repository programs.

## Supplementary Material

qxae031_Supplementary_Data

## Appendix 1: Detailed Methods section

We reviewed all state legislation in the United States regarding cancer drug repository programs. We first located the official online version of each state code. Utilizing a mix of search engine queries and queries within each state's official legislative website, we searched for the terms “drug” and “medication” in combination with “repository” and/or “recycling.” We then reviewed the relevant section of state codes that contained a statute addressing drug repository programs or medication recycling of any kind. We further queried the text of each statute for the terms “cancer” and “oncol[*]” to identify states with OACD-specific repository programs and recorded a list of such programs for further analysis.

The next step in our analysis was to determine whether states with legislation enabling or authorizing a drug repository program in fact had such a program in active operation. The applicable code section was searched to identify the state regulatory agency responsible for operating or overseeing the state's program. We then navigated to the website of the relevant state agency and reviewed the website to locate contact information for the program's coordinators.

Where an agency's website provided contact information such as a phone number or email, we contacted the program coordinators in order to verify whether the program was operational. If no such contact information was provided, an analysis of the agency's publicly available website was conducted, with primary attention to whether the website provided a list of participating pharmacies and/or healthcare systems. From this review, we compiled an internal database that recorded which states had passed legislation enabling drug repository programs, which state laws specifically provided for an OACD repository program, and which states were actively operating a repository program of any kind.

Our internal database was then compared with databases compiled by the National Conference of State Legislatures, the 2020 Position Statement on Drug Repository Programs published by the American Society for Clinical Oncology (ASCO), and Supporting Initiative to Redistributed Unused Medicine (SIRUM) to ensure accuracy of results.

Variables collected included program titles, date of inception, state act citation, regulating entity, number of participants, donor eligibility, recipient eligibility, cost to recipient, and ability to track outcomes. The above review was completed by the first author and was then reviewed by the remaining authors.
